# The influence of education on women’s well-being: Evidence from Australia

**DOI:** 10.1371/journal.pone.0247765

**Published:** 2021-03-24

**Authors:** Dai Binh Tran, Thao Dinh Ngoc Pham, Thuy Thanh Nguyen

**Affiliations:** Faculty of Economics and Management, Vietnamese-German University, Thủ Dầu Một, Binh Duong Province, Vietnam; The Open University, UNITED KINGDOM

## Abstract

This study investigates the relationship between women’s education and their level of well-being, using data from the Household, Income and Labor Dynamics in Australia (HILDA). To take into account potential endogeneity, the instrumental variables (IV) approach is employed, with partners’ education as an instrument. The findings show that higher education levels lead to a higher level of eudaimonic well-being, hedonic well-being, positive affect, and reduced psychological distress, highlighting a non-monetary benefit of education. Thus, policymakers should continue to widely promote education, in order for women to achieve higher levels of future well-being. Additionally, the findings show that the connection between education and well-being is mediated by healthy behaviors, such as engaging in physical activity, abstaining from drinking and smoking, social interactivity, and higher income. Therefore, public health campaigns which promote healthy behaviors among women should potentially mitigate gaps in formal education.

## Introduction

The present research was conducted to confirm the non-monetary benefits of education for women by examining the relationship between education and well-being among Australian women. According to statistics published in 2019, around 8.1% of young Australian women (aged 15–24 years) were not engaged in either work or study. Geographically, young Australians living outside major cities were less likely to be fully engaged in work and/or study, with 83 percent of young adults engaged in either employment or full-time education in major cities versus only 72 percent in outer regional and remote areas. For those not in school (aged 17–24 years), three-quarters were fully engaged in work and/or further study. Lastly, women aged 20 or above have been found to be less likely to engage in work and/or study than their male counterparts [1]. These figures show that a significant proportion of Australian women are not partaking in secondary or tertiary education.

A study by Mincer was the first to estimate the rate of returns to education [2]. Subsequently, a large number of studies have used a variety of different techniques to determine more precise estimations of the rate of returns to education [3–6]. However, the rate of returns to education may be underestimated when the non-monetary benefits of education are not taken into account [7]. Thus, more recently, economists have shifted their focus to the effect of education on many non-monetary outcomes, such as health [8], marriage [9], and subjective well-being [10].

The previous literature provides conflicting empirical evidence of how education affects people’s well-being. There are two contradictory findings regarding the relationship between education and well-being. First, it has been reported that education can have a significant positive impact on personal well-being. Findings based on the U.S. General Social Survey from 1972 to 2000 indicate that schools provide individuals with better decision-making skills, leading to better health, happier marriages, and more successful children [11]. Education has also been shown to increase patience and long-term thinking skills, trust, civic participation, and money management skills, and to reduce unhealthy behaviors, all contributing to greater happiness in life. For example, a study conducted to investigate the connection between education and happiness in Spain, using the European Social Survey, found that individuals with higher levels of education have a greater probability of being employed in a well-paid job, leading to higher levels of happiness [12]. It has also been found that, in Australia, people with a higher level of education are likely to have a higher level of well-being and life satisfaction [10].

In contrast, several research papers have suggested a negative relationship between education and well-being. For example, using data obtained from a survey of 5,000 British workers, it was found that people with a higher level of education are less satisfied with their life than people with a lower level of education, holding income constant [13]. In another study, using an Australian data set, the education levels of the reference group were found to be negatively correlated with individuals’ subjective well-being [14]. The negative effects of education on well-being can be explained by several factors. People with higher levels of education tend to have better-paid jobs, which involve more responsibilities and expectations, resulting in longer working hours, more stress, and reduced work-life balance [10]. Also, higher levels of education can make people more ambitious, with higher expectations that are more difficult to fulfill [13], which might reduce their levels of life satisfaction. It is also possible that some of their well-being is traded off, in exchange for an advanced career path [15]. Thus, given these contradictory results, there is a need for further research in order to obtain a more in-depth understanding of the link between education and well-being.

The conflicting results presented in previous literature create a demand for further research to gain more understanding and to enrich the current literature. Greater knowledge regarding this topic may contribute to improving future human capital development. In the process of evaluating an investment in education, policymakers can then take into account the value of well-being, which has hitherto been largely neglected. Additionally, women tend to bear more domestic responsibilities such as child nurturing. A previous study shows that more erudite women have a high tendency to spend more time playing with their children, highlighting another benefit of education of the mothers on their children’s development [8]. Thus, happier women become more significant for a more flourishing home life, in aspects such as childcare.

Several studies have been conducted to understand the mechanisms of how education affects well-being. For instance, education has been shown to directly influence happiness through building increasing self-confidence and self-estimation, and to indirectly affect happiness through income and labor status [12]. In another study, the positive relationship between education and life satisfaction was attributed to improvements in income and health [16]. Moreover, more highly educated women tend to exhibit healthier patterns of behaviors, including engaging in more physical activity and smoking less [8]. Also, since highly educated individuals tend to value their family and friendships more than less educated people [10], they tend to spend more time with friends and relatives, gathering more mental support. Lastly, income is also one of the key determinants of people’s satisfaction. Thus, the present study addresses the question of how education affects well-being by focusing on three key mechanisms: health behaviors, social interactions, and income.

The study focuses on women in Australia, firstly, as education among Australian women is consistently rising [17], with an increasing rate of returns to education [18]. Higher levels of education allow women to access more employment opportunities, rather than simply in traditional jobs commonly indicated as “female jobs” [8]. Secondly, as women tend to have more domestic responsibilities, including housework, household errands, or childcare [19], their state of well-being may involve more determining factors such as maternal health, which may provide the basis for further research on this topic.

The current study contributes to the literature from three different perspectives. Firstly, this study chooses Australian women as research subjects to reduce the heterogeneity and enrich the current literature on the topic in Australia. Secondly, the IV approach has been utilized to deal with the endogeneity of educational attainment levels, which has hitherto been largely overlooked in the previous literature. Lastly, where a relationship between education and well-being is shown to exist, the paper investigates the possible mechanisms of how education affects well-being to reveal potential policy implications for amplifying the positive effect of education on well-being.

The remainder of this paper is organized as follows. The next section provides an overview of the data source, along with a description of the key variables, the research model, and the estimation approach. The estimation results are then presented and discussed, followed by the conclusions of the study.

## Data description

This study utilizes data from Wave 2 to Wave 15 of the HILDA Survey, conducted during 2002–2015, and funded by the Department of Social Services of the Australian Government. This data was designed as panel data, gathering information of individuals from households, including their educational attainment level and well-being, which makes the dataset ideal to achieve the objectives of this study. Moreover, annual re-interview rates exceeded 95 percent in Wave 8 in 2008, and have remained above this level in subsequent waves [20], thereby minimizing the potential for attrition bias.

### Well-being measures

The dependent variables in this paper include six well-being measurements. The first is the evaluative measure, namely life satisfaction, which is the cognitive assessment of one’s own life. This item is defined with the following question "All things considered, how satisfied are you with your life?" The options for the answer range from 0, representing “totally dissatisfied” to 10, indicating “totally satisfied”.

The second well-being measurement is denoted as eudaimonic well-being, representing the evaluation of psychological well-being. Four questions were utilized from the dataset as follows: In the last four weeks, how often did you feel (1) worthless; (2) hopeless; (3) tired for no good reason; and (4) that everything in life was an effort. The answers are recorded as 1 (all of the time), 2 (most of the time), 3 (some of the time), 4 (a little of the time), and 5 (none of the time). The Cronbach’s alpha of the four questions is 0.80, indicating the questions are highly consistent, making it possible to average the responses to obtain a single index. A higher value of this index indicates a higher level of eudaimonic well-being. However, the data is only available in waves 7, 9, 11, 13, and 15, leading to a smaller sample for estimation.

The third measurement is the hedonic well-being, which assesses the psychological distress of the respondents. There are six questions recorded in the HILDA dataset as follows: In the last four weeks, about how often did you feel (1) so sad that nothing could cheer you up; (2) restless or fidgety; (3) nervous; (4) so restless that you could not sit still; (5) so nervous that nothing could calm you down and (6) depressed. The answers are from (1) all of the time to (5) none of the time. Cronbach’s alpha is 0.86, justifying the creation of an alternative measure by averaging six questions. The higher value of this index reflects a higher level of hedonic well-being.

Fourthly, the HILDA data set provides the Kessler Psychological Distress Scale Score as a measure of the level of well-being with the value ranging from 10 to 50. This measure is developed to determine the likelihood of having a mental disorder (e.g. psychological distress) [21]. When the scores are between 10 and 19, respondents are likely to be well. The range between 20 and 24 indicates that individuals are likely to have a mild disorder. Individuals are likely to have a moderate disorder if the scores are in the range of 25 to 29. The last group from 30 to 50 indicates that individuals have a severe disorder.

The final two measurements evaluate positive and negative affect. The questions regarding positive affect are as follows: How often within the past month? (1) Have you been happy; (2) Felt calm and peaceful; (3) Had a lot of energy; and (4) Felt full of life. Similarly, the negative affect is determined using five questions: How much of the time during the past four weeks? (1) Been nervous; (2) Felt down; (3) Felt worn out; (4) Felt tired; (5) Nothing could cheer you up. The recoded answers range from 1 “None of the time” to 6 “All of the time”. Given the Cronbach’s alpha is 0.87 for positive affect and 0.81 for negative affect, the positive affect index is taken as the average of the four answers and the negative affect index as the average of the five questions. We reverse the scores for positive and negative affect, so that a higher value of the index indicates a higher level of positive affect and negative affect.

### Education

Education is measured using years-of-schooling. The HILDA dataset derives the years-of-schooling variable from the highest level of educational attainment. The original question categorizes the highest level of educational attainment into groups such as master’s degree, graduate diploma or certificate, undergraduate degree, certificate III or IV, high school diploma, and below. Accordingly, the years of schooling are derived by the standard duration to complete each level of educational attainment level. For example, if respondents report that their level of educational attainment is a bachelor’s degree, their years of schooling are set as 15, combining twelve years of primary school, secondary school, high school, and three years of undergraduate studies.

### Potential mechanisms

Health behaviors, social interactions, and income are examined as potential mechanisms to mediate the effects of education on well-being. Smoking frequency is collected from the following question “Do you smoke cigarettes or any tobacco products?” The answers are from “I have never smoked or no longer smoke” to “I smoke daily”, representing the increasing smoking frequency. For drinking frequency, the respondents are asked “Do you drink alcohol?” The answers span from “I have never drunk alcohol or no longer drink alcohol” to “I drink every day”. The question regarding physical exercise is “In general, how often do you participate in moderate or intensive physical activity for at least 30 minutes?” Participants select their answers between 0 “not at all” and 5 “every day”. These self-reported measures are validated by medical studies in the previous literature [22, 23]. Empirically, they are also widely used in previous studies [24, 25]. For social contact measurement, the following question is asked: "In general, how often do you get together socially with friends and relatives not living with you”. The answers range from “Every day” to “Less often than once every 3 months”. Lastly, the logarithm of real annual disposable household income is used to represent income in this paper to make data more symmetric from the right-skewed distribution of income.

The data is extracted from Wave 2 to Wave 15 in the HILDA dataset since health behaviors are not available in Wave 1. The estimation sample is an unbalanced panel of 18,570 observations from 3,828 women after excluding observations with missing data. For ease of interpretation, the key variables, including well-being measures, health behaviors, and social contacts are standardized with mean 0 and standard deviation 1. Data is analyzed using the software package Stata 14.0. Descriptive statistics are shown in Table 1. This study requires no ethics approval for the authors as the analysis used only de-identified existing unit record data from the HILDA Survey. The authors had completed and signed the Confidentiality Deed Poll before the data applications’ approval. Therefore, datasets analyzed and/or generated during the current study are subject to the signed confidentiality deed.

**Table 1:** Descriptive statistics.

Variable	Observations	Mean	S.D.	Min	Max
Years of schooling	18,570	12.89	2.42	4	18.5
Husband’s years of schooling	18,570	12.87	2.38	4	19
Age	18,570	41.84	8.37	18	60
Homeowner	18,570	0.82	0.39	0	1
Real household income	18,570	112,409.10	74,253.53	562	952,142
Total of resident children	18,570	2.08	1.02	0	12
Total of non-resident children	18,570	0.45	0.92	0	12
**Health behaviors**					
Smoking	18,570	0.46	1.06	0	3
Drinking	18,570	2.20	1.71	0	6
Physical activities	18,570	2.37	1.46	0	5
Social contacts	18,570	3.29	1.43	0	6
**Evaluative well-being**					
Life satisfaction	18,570	7.97	1.27	0	10
**Eudaimonic well-being**	6,751	4.36	0.66	1	5
Worthless	6,751	4.69	0.70	1	5
Tired for no good reason	6,751	3.90	0.99	1	5
Everything an effort	6,751	4.20	0.90	1	5
Hopeless	6,751	4.64	0.70	1	5
**Hedonic well-being**	6,751	4.55	0.55	1	5
Feeling sad	6,751	4.71	0.64	1	5
Feeling restless or fidgety	6,751	4.43	0.78	1	5
Feeling nervous	6,751	4.19	0.83	1	5
Cannot sit still	6,751	4.75	0.61	1	5
Cannot calm down	6,751	4.79	0.56	1	5
Feeling depressed	6,751	4.43	0.82	1	5
**Positive affect**	18,570	4.02	0.96	1	6
Been a happy person	18,570	4.42	1.00	1	6
Felt calm and peaceful	18,570	3.86	1.18	1	6
Have a lot of energy	18,570	3.76	1.19	1	6
Feel full of life	18,570	4.06	1.13	1	6
**Negative affect**	18,570	2.37	0.80	1	6
Been a nervous person	18,570	1.99	1.04	1	6
Felt down	18,570	2.12	0.99	1	6
Felt worn out	18,570	2.89	1.14	1	6
Felt tired	18,570	3.27	1.16	1	6
Nothing could cheer you up	18,570	1.57	0.93	1	6
Psychology Distress Scale	6,751	15.24	5.64	10	50
**Marital status**					
Legally married	18,570	0.86	0.34	0	1
Living as a couple	18,570	0.14	0.34	0	1
Separated	18,570	0.00	0.02	0	1
Divorced	18,570	0.00	0.02	0	1
Single	18,570	0.00	0.01	0	1
**Employment status**					
Employed	18,570	0.76	0.42	0	1
Unemployed	18,570	0.02	0.15	0	1
Not in the labor force	18,570	0.21	0.41	0	1

## Methodology

The basic equation showing the relationship between education and well-being is:

$${WB}_{i}=\alpha X_{i}+\beta E_{i}+\varepsilon_{i}$$ (1)

where WB_i_ denotes the level of well-being (e.g. evaluative well-being, hedonic well-being, eudaimonic well-being, positive affect, negative affect and Psychology Distress Scale) of individual i; years of schooling are represented by E_i_; X_i_ represents the control variables including age, age squared, the number of children, home ownership status, employment status, marital status, waves, and regions; and ε_i_ is the error term.

However, one potential problem with E_i_ is endogeneity for several possible reasons. Firstly, reverse causality implies that happier students are likely to achieve higher academic success. For example, previous studies have found that students with a higher level of life satisfaction report more positive school experiences, greater participation in extracurricular activities, and higher GPAs than their less happy counterparts [26]. Secondly, schooling decisions may be explained by factors including motivation, ability, or family background of the individuals, which are unobservable, causing potential estimation bias. These issues are major methodological problems in this topic.

This paper employs the IV estimation method to tackle the endogeneity problem. Husband’s years of schooling is used as an instrumental variable for their wives’ years of schooling. This IV has been employed in the previous literature [27, 28]. The rationale for choosing the instrument is that a relationship between a wife’s education and her husband’s education potentially exists. Educated women are likely to choose partners having comparative education attainment levels since education is considered as a signaling device for a similar way of life, behaviors, social status, and economic outcomes [29]. Thus, the relationship of years of schooling between husbands and wives is estimated using the following equation in the first stage:

$$E_{i}=\alpha X_{i}+\theta {HE}_{i}+\varepsilon_{i}$$ (2)

where HE_i_ is the husband’s years of schooling. Then, Eq (1) becomes:

$${WB}_{i}=\alpha X_{i}+\beta {\hat{E}}_{i}+\varepsilon_{i}$$ (3)

where Ê_i_ represents the predicted women’s years of schooling obtained from Eq (2) and β represents the effect of education on subjective well-being. We also diagnose for multicollinearity by using the Variance Inflation Factor method. The results show that the value is lower than the threshold, showing that there is no multicollinearity in our estimation. In our estimation, robust standard errors clustered at the individual level are used.

## Results

### Ordinary least squares (OLS) regression

Eq (1) is estimated using the OLS approach. Evaluative well-being, hedonic well-being, eudaimonic well-being, positive affect, negative affect, and Kessler Psychological Distress Scale are dependent variables in the model. Other control variables are also included in the equation. The results in Table 2 indicate that an incremental year of schooling can improve eudaimonic well-being and hedonic well-being, while reducing the Psychological Distress Scale at *p*-value < 0.001. In terms of the magnitude of the coefficients, an additional year of schooling leads to an increase of 0.02 in standard deviations in both eudaimonic and hedonic well-being. Additionally, an additional year of schooling reduces the distress scale by 0.02 standard deviations at *p*-value < 0.001 and negative affect by 0.01 standard deviations at *p*-value < 0.01. However, this estimation technique might be biased because of the potential endogeneity of the education variable as mentioned above. Henceforth, the IV approach is presented in the subsequent section.

**Table 2:** Education and well-being with OLS estimation.

	Life satisfaction	Eudaimonic well-being	Hedonic well-being	Positive affect	Negative affect	Distress scale
Years of schooling	-0.00543	0.0270***	0.0268***	0.00493	-0.00991**	-0.0282***
	(0.0033)	(0.0052)	(0.0055)	(0.0032)	(0.0032)	(0.0054)
Age	-0.0458***	-0.00327	-0.0104	-0.0160*	0.0112	0.00785
	(0.0083)	(0.0137)	(0.0143)	(0.0082)	(0.0083)	(0.0141)
Age squared	0.0499***	0.00853	0.0167	0.0169	-0.0225*	-0.014
	(0.0100)	(0.0162)	(0.0168)	(0.0098)	(0.0099)	(0.0166)
Total of resident children	-0.0183	-0.0243	0.00613	-0.0172	0.00164	0.00744
	(0.0097)	(0.0154)	(0.0168)	(0.0093)	(0.0092)	(0.0163)
Total of non-resident children	0.0383**	-0.0319	-0.0384*	-0.0131	0.00931	0.0366
	(0.0135)	(0.0200)	(0.0195)	(0.0114)	(0.0113)	(0.0200)
Homeowner	0.204***	0.221***	0.247***	0.112***	-0.209***	-0.248***
	(0.0227)	(0.0363)	(0.0371)	(0.0218)	(0.0223)	(0.0368)
Unemployed	-0.197***	-0.389***	-0.461***	-0.146**	0.266***	0.455***
	(0.0568)	(0.1060)	(0.1080)	(0.0512)	(0.0557)	(0.1080)
Not in the labor force	0.0123	-0.140***	-0.169***	-0.146***	0.198***	0.166***
	(0.0213)	(0.0350)	(0.0354)	(0.0204)	(0.0208)	(0.0355)
Living as a couple	-0.0780***	-0.161***	-0.237***	-0.110***	0.158***	0.213***
	(0.0236)	(0.0390)	(0.0403)	(0.0234)	(0.0240)	(0.0398)
Separated	-1.437**	-0.4	-0.888	-0.847**	0.767*	0.712
	(0.5290)	(0.8230)	(1.0120)	(0.3160)	(0.3660)	(0.9690)
Divorced	-0.645	-	-	0.145	0.363	-
	(0.4590)	-	-	(0.5830)	(0.9100)	-
Single	0.294	0.873***	0.735***	-0.0306	0.42	-0.838***
	(0.4420)	(0.0601)	(0.0595)	(0.3790)	(0.4840)	(0.0596)
N	18,570	6,751	6,751	18,570	18,570	6,751

Note

* p<0.05

** p<0.01

*** p <0.001. Control variables include waves and residence regions. Robust standard errors are in parentheses.

### IV regression

The results of the first-stage regression are presented in Table 3. The table shows that a husband’s years of schooling correlate with his wife’s years of education, confirming the fact that the instrument of husbands’ years of schooling is a significant predictor of their wives’ education. This finding shows that an additional year of education of the husband equates with an increase of 0.42 years of additional schooling of their spouse. Moreover, the *F*-statistic is higher than the threshold value of 10 [30], rejecting the null hypothesis of a weak instrument. Thus, the IV approach is appropriated and will be applied for the rest of the estimation.

**Table 3:** First-stage regression.

	Years of schooling
Husband’s years of schooling	0.423***
	(0.0071)
Age	0.145***
	(0.0150)
Age squared	-0.180***
	(0.0186)
Total of resident children	-0.161***
	(0.0183)
Total of non-resident children	-0.360***
	(0.0257)
Homeowner	0.281***
	(0.0425)
Unemployed	-0.602***
	(0.0937)
Not in the labor force	-0.542***
	(0.0386)
Living as a couple	-0.0675
	(0.0475)
Separated	0.548
	(0.7010)
Divorced	0.0795
	(0.6990)
Single	-0.213
	(0.7000)
N	18,570

Note

* p<0.05

** p<0.01

*** p <0.001. Control variables include waves and residence regions. Robust standard errors are in parentheses.

Table 4 presents the results of the main estimation using the IV method. In comparison with the OLS, the IV approach delivers slightly different estimation results. Particularly, the effect of an extra year of schooling on positive affect becomes significant at *p*-value < 0.01, and the effect of education on negative affect becomes insignificant. Table 4 shows that there is a statistically significant positive effect of education on all four measures of well-being including eudaimonic well-being, hedonic well-being, and positive affect while reducing the psychological distress. This means that education has a desirable impact on women’s level of well-being, which shows a consistent result with previous literature [10–12]. Additionally, the magnitudes of coefficients across all the well-being measures are higher than those in Table 2, indicating the downward bias of the OLS approach. Table 4 also reveals other important determinants of women’s well-being. For example, home ownership plays an essential role in determining women’s well-being. Moreover, if the respondents are unemployed, all of their well-being measures are significantly reduced. Lastly, the marital status of living as a couple (de facto) also reduces well-being among women.

**Table 4:** Education and well-being with IV estimation.

	Life satisfaction	Eudaimonic well-being	Hedonic well-being	Positive affect	Negative affect	Distress scale
Years of schooling	0.0137	0.0645***	0.0417***	0.0238**	-0.0111	-0.0547***
	(0.0079)	(0.0128)	(0.0126)	(0.0076)	(0.0076)	(0.0127)
Age	-0.0500***	-0.0137	-0.0145	-0.0202*	0.0115	0.0152
	(0.0085)	(0.0141)	(0.0147)	(0.0083)	(0.0085)	(0.0145)
Age squared	0.0548***	0.0206	0.0214	0.0217*	-0.0228*	-0.0225
	(0.0102)	(0.0166)	(0.0172)	(0.0100)	(0.0101)	(0.0170)
Total of resident children	-0.0139	-0.0141	0.0102	-0.0129	0.00137	0.00025
	(0.0098)	(0.0154)	(0.0169)	(0.0094)	(0.0093)	(0.0164)
Total of non-resident children	0.0490***	-0.0106	-0.0299	-0.00263	0.00866	0.0215
	(0.0141)	(0.0210)	(0.0205)	(0.0121)	(0.0120)	(0.0210)
Homeowner	0.195***	0.204***	0.240***	0.103***	-0.208***	-0.235***
	(0.0229)	(0.0366)	(0.0374)	(0.0219)	(0.0225)	(0.0371)
Unemployed	-0.182**	-0.358***	-0.449***	-0.132*	0.265***	0.433***
	(0.0571)	(0.1060)	(0.1080)	(0.0515)	(0.0559)	(0.1080)
Not in the labor force	0.0227	-0.122***	-0.162***	-0.136***	0.198***	0.153***
	(0.0215)	(0.0356)	(0.0358)	(0.0206)	(0.0211)	(0.0359)
Living as a couple	-0.0712**	-0.146***	-0.231***	-0.103***	0.157***	0.203***
	(0.0238)	(0.0390)	(0.0403)	(0.0236)	(0.0240)	(0.0398)
Separated	-1.443**	-0.457	-0.911	-0.852**	0.768*	0.753
	(0.5270)	(0.7840)	(0.9930)	(0.3130)	(0.3650)	(0.9390)
Divorced	-0.637	-	-	0.154	0.363	-
	(0.4480)	-	-	(0.5770)	(0.9090)	-
Single	0.312	0.810***	0.709***	-0.013	0.419	-0.793***
	(0.4270)	(0.0634)	(0.0627)	(0.3650)	(0.4830)	(0.0628)
N	18,570	6,751	6,751	18,570	18,570	6,751

Note

* p<0.05

** p<0.01

*** p <0.001. Control variables include waves and residence regions. Robust standard errors are in parentheses.

Table 5 shows the results of the robustness check using alternative instruments including parent’s education. This instrument is widely used in the previous literature [6, 31]. One study suggests that highly educated parents have a higher expectation for academic performance, which is likely to influence their children’s level of education [32]. In other words, these children are likely to seek to obtain more education to meet their parents’ expectations. Notably, parent’s education is collected from Wave 6, leading to a smaller sample, which accounts for 13,353 observations. Using the alternative instruments, the results remain consistent for eudaimonic well-being, hedonic well-being, positive affect, and distress scale, strengthening the previous findings.

**Table 5:** Robustness check—alternative instruments.

	**Father’s education as the instrument variable**					
	Life satisfaction	Eudaimonic well-being	Hedonic well-being	Positive affect	Negative affect	Distress scale
Years of schooling	0.0277	0.0613**	0.0621**	0.0494***	-0.0414**	-0.0648**
	(0.0145)	(0.0203)	(0.0205)	(0.0143)	(0.0142)	(0.0204)
N	13,353	6,751	6,751	13,353	13,353	6,751
	**Mother’s education as the instrument variable**					
	Life satisfaction	Eudaimonic well-being	Hedonic well-being	Positive affect	Negative affect	Distress scale
Years of schooling	0.0642***	0.153***	0.127***	0.0796***	-0.0927***	-0.146***
	(0.0158)	(0.0229)	(0.0231)	(0.0156)	(0.0157)	(0.0232)
N	13,353	6,751	6,751	13,353	13,353	6,751

Note: * p<0.05

** p<0.01

*** p <0.001. Control variables include age, age squared, total resident/non-resident children, a dummy of homeowner and dummies of employment status, marital status, waves and residence regions. Robust standard errors are in parentheses.

### Education and health behaviors, social interactions, and income

Firstly, people with higher levels of education have the tendency to practice more healthy behaviors such as engaging in more physical exercise, and less smoking or drinking [33], leading to a higher level of well-being. From the first three columns in Table 6, it can be seen that more engagement in physical activities and less smoking are common to women with higher levels of education, which is consistent with our expectation. Since the variable of drinking in the estimation measures the frequency of drinking, but not the alcohol amount consumed, though the results indicate a positive relationship between education and drinking, it is possible that frequent consumption of a moderate amount of alcohol may provide some health benefits such as a reduced risk of heart disease, ischemic stroke, and diabetes [34]. Secondly, one study indicates that more social interactions reduce the feeling of loneliness and lack of support, leading to a positive impact on their state of mind [35]. To test this hypothesis, we use the frequency of visiting friends and/or non-resident relatives as a dependent variable to proxy for social support. The results shown in Table 6 reveal that more highly educated women have a greater frequency of socializing with friends and non-resident relatives. Lastly, income is a potential mechanism whereby education can affect people’s well-being. The findings from the last column in Table 6 show that more highly educated individuals are more likely to have a higher household income than their less educated counterparts, which confirms the positive rate of return to education among women.

**Table 6:** Education and health behaviors, social contacts and income.

	Smoking	Drinking	Physical activities	Social contacts	Household income
Years of schooling	-0.162***	0.0846***	0.0215**	0.0531***	0.122***
	(0.0069)	(0.0075)	(0.0075)	(0.0076)	(0.0039)
Age	0.0114	0.0669***	-0.0144	-0.0750***	0.00167
	(0.0089)	(0.0074)	(0.0083)	(0.0084)	(0.0041)
Age squared	-0.0274**	-0.0599***	0.0267**	0.0643***	0.0154**
	(0.0105)	(0.0091)	(0.0100)	(0.0101)	(0.0050)
Total of resident children	-0.0119	-0.0190*	-0.00421	-0.0354***	0.0765***
	(0.0091)	(0.0088)	(0.0091)	(0.0097)	(0.0045)
Total of non-resident children	0.0157	-0.0171	0.00714	0.0380**	0.00766
	(0.0122)	(0.0115)	(0.0118)	(0.0128)	(0.0064)
Homeowner	-0.199***	0.0707***	-0.0072	0.111***	0.116***
	(0.0232)	(0.0204)	(0.0216)	(0.0213)	(0.0107)
Unemployed	0.0525	-0.175***	0.0154	-0.0986	-0.180***
	(0.0544)	(0.0458)	(0.0490)	(0.0529)	(0.0257)
Not in the labor force	-0.110***	-0.226***	-0.0158	0.164***	-0.109***
	(0.0190)	(0.0185)	(0.0200)	(0.0198)	(0.0101)
Living as a couple	0.466***	0.277***	0.00509	-0.0452	0.0226*
	(0.0272)	(0.0220)	(0.0231)	(0.0232)	(0.0107)
Separated	0.0146	-0.405	-0.0389	-0.493	-0.0678
	(0.2300)	(0.2290)	(0.3480)	(0.3710)	(0.1250)
Divorced	-0.448**	0.102	0.983**	-0.288	-0.0296
	(0.1620)	(0.4650)	(0.3020)	(0.7200)	(0.2670)
Single	-0.000515	0.172	-0.17	-0.295	0.434
	(0.6100)	(0.3470)	(0.5890)	(0.4910)	(0.4010)
N	18,570	18,570	18,570	18,570	18,570

Note

* p<0.05

** p<0.01

*** p <0.001. Control variables include waves and residence regions. Robust standard errors are in parentheses.

In conclusion, education is found to have a statistically significant impact on health behaviors, social interactions, and income. The next section will examine whether changes in those factors lead to the improvement of well-being.

### Mediation analysis

To confirm the mediating role, we re-estimate the Eq (3), and proposed mediators are included as additional regressors. There are two requirements that need to be met. First, the coefficients of the effect of education on well-being are expected to drop when these mediators are controlled. Second, the effects of mediators on well-being must remain statistically significant [36]. The results are presented in Table 7, from which it can be seen that the coefficients of education become insignificant for some of the measurements of well-being including hedonic well-being, positive affect, and distress scale, indicating the case of perfect mediation once the mediators are controlled. Moreover, the size of the effect drops when the mediators are included in specification 2. For example, the effect of education on life satisfaction reduces by 272 percent. Similarly, there is a reduction in the effect on eudaimonic well-being, hedonic well-being, positive affect, negative affect, and distress scale, dropping by 52 percent, 99 percent, 103 percent, 306 percent, and 73 percent respectively. Then, the Sobel test [37] is employed to test the significance of a mediation effect. The results show that almost all of the coefficients are statistically significant including smoking, physical activities, social contacts, and income, satisfying the above-mentioned requirements, except for drinking behaviors. Thus, it can be concluded that the positive effect of education on well-being can be explained by healthy habits, the extent of social capital, and higher income.

**Table 7:** Mediation analysis.

	Life satisfaction		Eudaimonic		Hedonic		Positive affect		Negative affect		Distress scale	
	(1)	(2)	(1)	(2)	(1)	(2)	(1)	(2)	(1)	(2)	(1)	(2)
Years of schooling	0.0137	-0.0235**	0.0645***	0.0307*	0.0417***	0.000559	0.0238**	-0.000713	-0.0111	0.0229**	-0.0547***	-0.0149
	(0.0079)	(0.0090)	(0.0128)	(0.0146)	(0.0126)	(0.0145)	(0.0076)	(0.0085)	(0.0076)	(0.0087)	(0.0127)	(0.0145)
Smoking		-0.0562***		-0.0735***		-0.0978***		-0.0479***		0.0695***		0.0914***
		(0.0092)		(0.0159)		(0.0165)		(0.0084)		(0.0089)		(0.0163)
		[5.9245]		[4.5358]		[5.7475]		[5.5480]		[7.4486]		[5.4544]
Drinking		0.0146		-0.00604		0.00195		-0.0241**		-0.0244**		0.00231
		(0.0076)		(0.0125)		(0.0129)		(0.0076)		(0.0076)		(0.0127)
		[1.8939]		[0.4828]		[0.1511]		[3.0567]		[3.0920]		[0.1819]
Physical activities		0.148***		0.174***		0.121***		0.238***		-0.178***		-0.153***
		(0.0076)		(0.0125)		(0.0125)		(0.0074)		(0.0075)		(0.0125)
		[2.8324]		[2.8042]		[2.7453]		[2.8517]		[2.8424]		[2.7876]
Social contacts		0.134***		0.156***		0.149***		0.151***		-0.133***		-0.161***
		(0.0080)		(0.0136)		(0.0141)		(0.0077)		(0.0078)		(0.0139)
		[6.4604]		[5.9728]		[5.8335]		[6.5931]		[6.4724]		[5.9885]
Real household income		0.136***		0.0670*		0.104***		0.0462**		-0.0803***		-0.0921**
		(0.0188)		(0.0288)		(0.0291)		(0.0170)		(0.0174)		(0.0288)
		[7.0499]		[2.3200]		[3.5510]		[2.7076]		[4.5660]		[3.1815]
Age	-0.0500***	-0.0383***	-0.0137	0.00344	-0.0145	0.000935	-0.0202*	-0.00331	0.0115	-0.000101	0.0152	-0.00194
	(0.0085)	(0.0083)	(0.0141)	(0.0137)	(0.0147)	(0.0145)	(0.0083)	(0.0080)	(0.0085)	(0.0083)	(0.0145)	(0.0142)
Age squared	0.0548***	0.0395***	0.0206	0.0000199	0.0214	0.00224	0.0217*	0.00213	-0.0228*	-0.00778	-0.0225	-0.00156
	(0.0102)	(0.0100)	(0.0166)	(0.0161)	(0.0172)	(0.0170)	(0.0100)	(0.0096)	(0.0101)	(0.0098)	(0.0170)	(0.0166)
Total of resident children	-0.0139	-0.0193*	-0.0141	-0.0162	0.0102	0.00535	-0.0129	-0.0111	0.00137	0.00243	0.00025	0.00403
	(0.0098)	(0.0097)	(0.0154)	(0.0151)	(0.0169)	(0.0168)	(0.0094)	(0.0090)	(0.0093)	(0.0092)	(0.0164)	(0.0161)
Total of non-resident children	0.0490***	0.0429**	-0.0106	-0.0124	-0.0299	-0.0309	-0.00263	-0.0101	0.00866	0.0141	0.0215	0.0229
	(0.0141)	(0.0137)	(0.0210)	(0.0196)	(0.0205)	(0.0194)	(0.0121)	(0.0115)	(0.0120)	(0.0116)	(0.0210)	(0.0196)
Homeowner	0.195***	0.153***	0.204***	0.155***	0.240***	0.184***	0.103***	0.0748***	-0.208***	-0.170***	-0.235***	-0.179***
	(0.0229)	(0.0224)	(0.0366)	(0.0358)	(0.0374)	(0.0369)	(0.0219)	(0.0209)	(0.0225)	(0.0219)	(0.0371)	(0.0363)
Unemployed	-0.182**	-0.141*	-0.358***	-0.340***	-0.449***	-0.421***	-0.132*	-0.114*	0.265***	0.232***	0.433***	0.409***
	(0.0571)	(0.0557)	(0.1060)	(0.1020)	(0.1080)	(0.1050)	(0.0515)	(0.0487)	(0.0559)	(0.0558)	(0.1080)	(0.1050)
Not in the labor force	0.0227	0.015	-0.122***	-0.156***	-0.162***	-0.190***	-0.136***	-0.163***	0.198***	0.210***	0.153***	0.185***
	(0.0215)	(0.0211)	(0.0356)	(0.0340)	(0.0358)	(0.0347)	(0.0206)	(0.0197)	(0.0211)	(0.0205)	(0.0359)	(0.0344)
Living as a couple	-0.0712**	-0.0468*	-0.146***	-0.111**	-0.231***	-0.188***	-0.103***	-0.0698**	0.157***	0.128***	0.203***	0.161***
	(0.0238)	(0.0234)	(0.0390)	(0.0380)	(0.0403)	(0.0396)	(0.0236)	(0.0226)	(0.0240)	(0.0236)	(0.0398)	(0.0389)
Separated	-1.443**	-1.355*	-0.457	-0.329	-0.911	-0.767	-0.852**	-0.775**	0.768*	0.679	0.753	0.61
	(0.5270)	(0.5530)	(0.7840)	(0.6780)	(0.9930)	(0.9340)	(0.3130)	(0.2970)	(0.3650)	(0.3560)	(0.9390)	(0.8560)
Divorced	-0.637	-0.767	-	-	-	-	0.154	-0.0549	0.363	0.53	-	-
	(0.4480)	(0.4380)	-	-	-	-	(0.5770)	(0.5710)	(0.9090)	(0.8970)	-	-
Single	0.312	0.315	0.810***	0.565***	0.709***	0.572***	-0.013	0.0562	0.419	0.389	-0.793***	-0.597***
	(0.4270)	(0.3840)	(0.0634)	(0.0709)	(0.0627)	(0.0712)	(0.3650)	(0.2560)	(0.4830)	(0.4290)	(0.0628)	(0.0708)
Observation	18,570	18,570	6,751	6,751	6,751	6,751	18,570	18,570	18,570	18,570	6,751	6,751

Note

* p<0.05

** p<0.01

*** p <0.001. Control variables include waves and residence regions. Robust standard errors are in parentheses. Sobel tests are presented in square bracket.

## Conclusion

This study examines the effect of women’s education on well-being using the HILDA dataset. After addressing the endogeneity problem of education by using the IV approach, the findings suggest that education can improve an individual’s level of well-being, including eudaimonic well-being, hedonic well-being, positive affect, and a reduction in Psychological Distress Scale. Also, this relationship is mediated by physical activities, reduced smoking, social interactions, and higher income.

Nevertheless, this research has limitations. This study has not taken into account the effects among men, which might be different from those among women, reducing the generalizability of the findings. Additionally, this study uses the subjective measurement of well-being. Thus, several objective measurements of well-being could be used to explore other aspects of well-being, which education may affect.

To promote a higher level of well-being among women in the future, several policy implications can be considered to (1) encourage women to obtain a higher level of education and (2) catalyze the positive relationship. Based on the current statistics presented in the introduction section, policymakers should continue to facilitate women’s and young girls’ education through particular projects or interventions. For example, greater access to schools for female students should be highly supported, by raising awareness of the importance of early childhood education among parents and families. This policy is especially crucial in remote areas, where limited access to education still exists. For higher levels of education, although there is an increase in labor force participation and increased women’s levels of education, this can be improved by creating more favorable conditions for women such as flexible working arrangements, childcare support, and parental leave policies. Additionally, gender pay gap is also a problem with Australian women. According to the Workplace Gender Equality Agency [38], Australia’s national gender pay gap is 14 percent in 2020, 24.1 percent for professional, scientific, and technical services, which lowers the value of education of women. The precise returns to education of women should be critically re-evaluated.

Lastly, the complete mediation of the education and well-being relationship by healthy behaviors strongly suggests that gaps in formal education can be mitigated by efforts to increase healthy behaviors, and therefore investment in public health campaigns is strongly recommended. For example, smoking and alcohol consumption prevention campaigns should be promoted. Moreover, frequent physical activity should be endorsed or incorporated into school curricula to advocate women to have more active lives, thereby catalyzing the benefits of education on well-being.
